# Non-fatal injuries among pediatric patients seeking care in an urban Ghanaian emergency department

**DOI:** 10.1186/1865-1380-5-36

**Published:** 2012-09-26

**Authors:** Lauren K Whiteside, Rockefeller Oteng, Patrick Carter, John Amuasi, Ekua Abban, Sarah Rominski, Michelle Nypaver, Rebecca M Cunningham

**Affiliations:** 1 Department of Emergency Medicine, University of Michigan, UM Injury Center 24 Frank Lloyd Wright Drive Suite H3200, Ann Arbor, MI, 48105, United States of America; 2 Komfo Anokye Teaching Hospital (KATH), P.O. Box KS 1934, Kumasi, Ghana; 3 Division of Health Policy and Management, University of Minnesota School of Public Health, 420 Delaware Street S.E, Minneapolis, MN, 55455, United States of America; 4 School of Medicine University of Michigan, 1301 Catherine Street, Ann Arbor, MI, 48109, United States of America; 5 Global REACH, University of Michigan, 1301 Catherine Street, Ann Arbor, MI, 48109, United States of America; 6 Department of Emergency Medicine/Children’s Emergency Services, University of Michigan, 1500 E. Medical Center Drive, Ann Arbor, MI, 48109-5305, United States of America

## Abstract

**Background:**

According to the World Health Organization (WHO), injuries represent the largest cause of death among people ages 140 –and contribute to a large burden of disease worldwide. The aims of this study were to characterize the prevalence and relative mechanism of injury among children seeking emergency care and describe the demographics at time of presentation among these children to inform further research in Ghana and sub-Saharan Africa.

**Methods:**

A prospective cross-sectional survey of pediatric patients (*n* = 176) was conducted between 13 July 2009 and 30 July 2009 in the Accident and Emergency Center at Komfo Anoche Teaching Hospital (KATH) in Kumasi, Ghana. Participants were asked questions regarding demographics, insurance status, overall health, and chief complaint.

**Results:**

Of the 176 patients surveyed, 66% (*n* = 116) presented for injuries. The mean age was 4.7 years (range 1.5 months to 17 years), and 68% (*n* = 120) were male. Of those presenting with injury, 43% (*n* = 50) had road traffic injuries (RTI). Of the RTIs, 58% (*n* = 29) were due to being an occupant in a car crash, 26% (*n* = 13) were pedestrian injuries, and 14% (*n* = 7) were from motorcycles. There was no significant difference in demographics, health status or indicators of socioeconomic status between injured and non-injured patients.

**Conclusions:**

Among pediatric patients presenting for acute care at KATH during the study time frame, the majority (*n* = 116, 66%) presented for injuries. To date, there are no studies that characterize pediatric patients that present for acute care in Ghana. Identifying injury patterns and collecting epidemiologic data are important to guide future research and educational initiatives for Emergency Medicine.

## Background

Injury represents a major and increasing global health concern. Historically, infectious diseases have accounted for a large majority of childhood morbidity in low and middle income countries (LMICs). This trend is transitioning, and according to the World Health Organization (WHO), injuries are now the leading cause of death among people ages 140 –[1]. In 2004, injury caused more deaths than HIV, malaria and tuberculosis combined [2]. The WHO reports that 950,000 deaths a year in people less than 18 years of age are attributable to injuries, with 95% of these deaths occurring in LMICs [1].

Specifically, the most common cause of death for children ages 15–19 worldwide is road traffic injuries. In low-income regions such as Africa, a large number of traffic-related deaths are among pedestrians, passengers, cyclists, users of motorized two-wheelers, and occupants of buses and minibuses [3,4]. LMICs, including Ghana, have roads that carry a wide variety of road traffic from pedestrians and bicyclists to heavy goods commercial vehicles without any physical separation. Children and elderly pedestrians are often the most vulnerable road users, and, according to the WHO, Africa has the highest mortality rate from road traffic injuries worldwide at 28.3 per 100,000 population. In comparison, the US has a mortality rate of 15.2 per 100,000 population due to road traffic injuries [5].

Within West Africa, Ghana has historically been a leader in change and industrialization. Ghana was the first sub-Saharan country to achieve independence from colonial rule in 1957 and has continued to lead the way in this region in government stability, economic growth, and improved efforts to reduce morbidity and mortality due to injury. The starting point for injury surveillance is often mortality data. Injuries are the sixth leading cause of death in children under 5 years of age in Ghana; however, there are no further data regarding how these children were injured [6]. Additionally, data are lacking on the number of non-fatal injuries in this population. A post-mortem study on children aged 10–19 who died from injury in Accra, Ghana, revealed drowning to be the number one cause of mortality (37%) followed closely by road traffic accidents (34%). The authors note that pedestrians hit by a car accounted for 70% of road traffic injury deaths in this sample (*n* = 151) [7]. Although understanding mortality is crucial, mortality represents the tip of the iceberg for injury prevention. There is currently a paucity of detailed data on non self-report, non-fatal pediatric injury severe enough to require medical attention. In order to assess the potential impact, need, and type of pediatric injury prevention strategies needed in Ghana, a clear assessment of non-fatal injury requiring medical attention among children is needed.

The aims of this study were to: (1) define the prevalence of pediatric injury among children seeking emergency care; (2) characterize the mechanism of injury responsible for the need for medical treatment, and (3) describe the demographic characteristics and health status at time of presentation among children seeking care for injury. The findings from this study will inform future injury prevention and acute trauma care research in Ghana and countries within Africa.

## Methods

### Study setting

A prospective cross-sectional survey of pediatric patients seeking emergency care was conducted. Potential participants included all patients seeking care in the Accident and Emergency (A&E) Center at Komfo Anokye Teaching Hospital (KATH) in Kumasi who were able to give informed consent. Inclusion criteria were age under 18 years presenting to KATH A&E between 13 July 2009 and 30 July 2009 during study hours. The study site, KATH, is located in Kumasi, Ghana, which is the second largest city in the country with a population of approximately 1.17 million at the 2000 census [8]. Study procedures were approved and conducted in compliance with the Komfo Anokye Teaching Hospital, School of Medical Sciences, Kwame Nkrumah University of Science and Technology Committee on Human Subjects, Publication and Ethics and the University of Michigan Institutional Review Board for Human Subjects guidelines.

The A&E center opened in May 2009 and serves Kumasi and the surrounding area. This facility is open 24 h a day and in 2009 was treating approximately 25,000 adult and pediatric patients per year. The entire KATH Hospital utilizes more than 800 hospital beds and serves as the principal referral hospital for the northern two thirds of Ghana [9]. Patients arrive at the A&E center by private car, public transportation, or ambulance.

### Recruitment

Questionnaires were administered to the parents of children under 18 after parental consent or, if children were old enough to complete the survey on their own (i.e., age over 14), with parental consent and child assent. Recruitment proceeded systematically during 8-h shifts (7 a.m.-3 p.m., 3 p.m.-11 p.m., 11 p.m.-7 a.m.) designed for sampling over days, evenings, nights, and weekends. Data collection took place between 13 July and 30 July 2009. The survey was administered in the local languages of Twi or Fante or in English. Patients were excluded if a parent was unavailable to provide consent (*n* ~ 22), if unable to speak Twi, Fante, or English if translation for Twi or Fante was not available (*n* ~ 16), if the patient had an altered mental status (*n* ~ 19), if the patient was in need of acute care (e.g., transfer to the operating room, placement on a ventilator) precluding survey response (*n* ~ 13), if the patient was sedated (*n* ~ 75), or if they were admitted, transferred, or expired before survey completion (*n* ~ 13). Further data on excluded patients including chief complaint were not obtained. Each survey was administered by trained research staff and took approximately 10–15 min to complete. Surveys were administered while patients were waiting to be triaged, waiting for care after triage, or after care had been initiated by the physician.

### Survey content and administration

Measures of demographics and overall health were drawn from the Ghana National Survey [10,11]. The mobility status of each participant was ascertained as a marker of illness and injury severity. Patients were also asked if they participated in the Ghana National Health Insurance Scheme 12. The socioeconomic index was constructed based on measures obtained from the Ghana National Survey [10,11].

Research assistants (RA) also collected data from subjects on their chief complaint and mobility. At the time of the survey, vital signs were not routinely part of triage care, and documentation of disposition following their visit to the A&E center was not tracked systematically in this facility; this information is therefore not available to assess the severity of illness or injury. The RAs’ assessments of mobility and mental status were recorded as proxy measures of severity. Research staff was trained on the classification of injury by the ICD-9 E-code [12]. Injury visits were classified as intentional (E950–E969) or unintentional (E800–E869, E880–E929) by the RAs based on patient interview at the time of the visit.

### Calculations

Data were analyzed using SPSS version 17 (SPSS Inc, Chicago, IL). Descriptive statistics *n* (%)] of key variables were computed, and bivariate analysis comparing the injured population to the non-injured population was conducted. Using principal component analysis (PCA) [13], the following five items comprised the Socioeconomic Index: ‘Does your household have electricity? Does your household have a radio? Does your household have a video deck/DVD/VCR? Does your household have a phone? Does your household have a refrigerator?’ The socioeconomic index was dichotomized into “poor” (lower 40% of the index) versus “not poor” (upper 60% of the index).

## Results

During the study period, 176 surveys were completed (~72% participation rate). Injury represented 66% (*n* = 116) of all A&E visits in this pediatric population (up to 18 years of age). Approximately two-thirds (*n* = 120) of the respondents were male. The mean age was 4.7 years with a range of 1.5 months to 17 years old, and 75% (*n* = 132) of the patients were less than 5 years of age. Over 80% (*n* = 143) of all participants had a parent that worked outside of the home, and 57% (*n* = 101) of parents had completed secondary or middle school. There were no statistically significant differences in demographic variables between those that presented for injury-related complaints and those that had non-injury-related complaints (Table 1).

**Table 1:** **Demographics and health status,*n*= 176**

**Characteristics**	**All patients*n*= 176,*n*(%)/M(SD)**	**Injured patients*n*= 116,*n*(%)/M(SD)**	Non-injured patients,**n= 57,*n*(%)/M(SD)	**OR and 95 % CI**
**Demographics**				
Male	120 (68.2)	78 (67.2)	41 (71.98)	0.80 (0.40, 1.61)
Age (years)	4.7(4.7)	4.4 (4.0)	4.8 (5.7)	
< 5 years old	132 (75.0)	87 (75.0)	45 (78.9)	0.80 (0.37, 1.72)
5-11 years old	21 (11.9)	19 (16.4)	1 (1.8)	
12-18 years old	23 (13.1)	10 (8.6)	11 (19.3)	
**Parental education**				
Completed secondary school	39 (22.6)	22 (19.3)	16 (29.1)	0.55 (0.26, 1.18)
Completed middle school	62 (36.0)	39 (34.2)	22 (40.0)	
Completed primary school	43 (25.0)	33 (28.9)	10 (18.2)	
Parents employed out of the house	143 (81.7)	98 (85.2)	43 (75.4)	1.88 (0.85, 4.15)
**Characteristics of ED acute care visit**				
**Mode of transportation to A&E**				
Private car	95 (54.0)	61 (54.0)	33 (60.0)	
Public transportation	37 (21.0)	24 (21.2)	12 (21.8)	
Ambulance	33 (18.8)	24 (21.2)	9 (16.3)	1.38 (0.59, 3.21)
Other	5 (2.9)	4 (3.6)	1 (1.8)	
Traveled 1 h or more	77 (51.0)	47 (48.0)	28 (54.9)	0.76 (0.38, 1.49)
**Mobility**				
Ambulatory (or developmentally not walking)*	100 (58.5)	57 (49.0)	40 (74.1)	
Required a stretcher, walking with help	71 (42.5)	57 (50.0)	14 (25.9)	0.35 (0.17, 0.71)^1^
**Past-year health**				
Very good	112 (82.4)	97 (84.3)	12 (66.7)	
Good	19 (14.0)	14 (12.2)	5 (27.8)	2.69 (0.90, 8.11)
Moderate, bad, or very bad	5 (3.7)	4 (3.4)	1 (5.6)	
Insurance (national or other) (yes)	132 (81.5)	87 (81.3)	42 (80.8)	1.04 (0.44, 2.41)

*Infants who were not ambulatory.

**n = 3 missing reason for visit.

^1^*p* < 0.05.

The majority of people who arrived to the A&E center (54%, *n* = 95) were transported by private car, while 21% (*n* = 37) arrived by bus (tro-tro) or public transportation; only 19% (33 patients) arrived by ambulance. Over 50 percent of all participants (*n* = 77) traveled more than 60 min to get to KATH (*n* = 88), and 41% (*n* = 47) of injured patients traveled more than 1 h to seek care. As a marker of illness severity, ambulatory status was obtained. Of those patients who presented for injury, 50% (*n* = 57) were ambulatory or developmentally not walking, and 50% (*n* = 57) required a stretcher or were immobile (data missing on two participants). Those who presented for injury-related complaints were less likely to be ambulatory (OR 0.35, 95% CI 0.17-0.71) compared to those with medical or non-injury related complaints.

Socioeconomic markers are listed in Table 2. A large majority of people surveyed reported having electricity (87%, *n* = 153), a radio (84%, *n* = 148), a phone (78%, *n* = 138), and a television (75%, *n* = 132). Additionally, two thirds of respondents (68%) reported having a refrigerator (*n* = 119) at home. In contrast, less than 20% of respondents owned a car (*n* = 33), a bicycle (*n* = 17), or a motorcycle (*n* = 13). Also, less than half (45%) of respondents had a flush toilet at home (*n* = 79). There was no difference in the socioeconomic index between injured and non-injured patients.

**Table 2:** Socioeconomic status indicators

**Socioeconomic status indicators**	**All patients*n*= 176,*n*(%)**	**Injured patients*n*= 116, n(%)**	**Non-injured patients*n*= 57,*n*(%)**	OR (95% CI)**
Flush toilet	79 (45.4)	52 (45.2)	27 (48.2)	0.89 (0.47, 1.68)
Electricity	153 (87.4)	100 (87.0)	52 (91.2)	0.64 (0.22, 1.86)
Radio	148 (84.6)	97 (84.3)	48 (84.2)	1.01 (0.42, 2.42)
Television	132 (75.4)	89 (77.4)	41 (71.9)	1.34 (0.65, 2.76)
DVD/video deck	120 (69.0)	84 (73.7)	34 (59.6)	1.89 (0.97, 3.71)
Phone	138 (78.9)	92 (80.0)	44 (77.2)	1.18 (0.55, 2.55)
Refrigerator	119 (68.0)	85 (73.9)	33 (57.9)	2.06 (1.05, 4.03)
Bicycle	17 (9.7)	7 (6.1)	9 (15.8)	0.35 (0.12, 0.98)^1^
Motorcycle/scooter	13 (7.4)	10 (8.7)	3 (5.3)	1.71 (0.45, 6.49)
Car/truck	33 (18.9)	24 (20.9)	8 (14.0)	1.61 (0.67, 3.86)
**Socioeconomic index**				
Poor	70 (39.8)	40 (34.5)	28 (49.1)	0.54 (0.29, 1.04)
Not poor	106 (60.2)	76 (65.5)	29 (50.9)	

^1^*p* < 0.05.

**Compares injured to non-injured patients.

**Figure 1:**
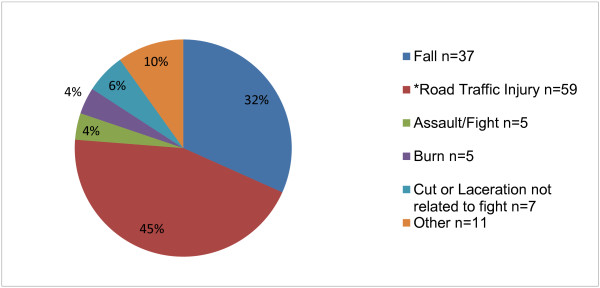
Types of injury.

**Figure 2:**
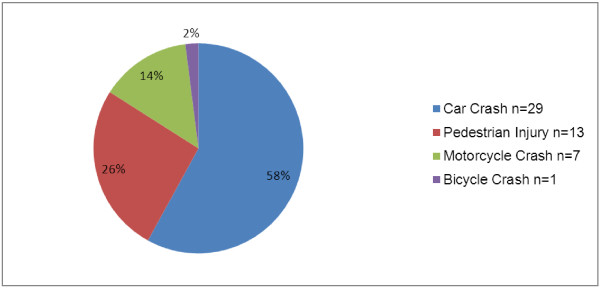
Road traffic injuries.

The mechanism of injury for all patients seeking care for injury is depicted in Figure 1. Road traffic injuries accounted for 43% (*n* = 50) of injured patients. Of the road traffic injuries (Figure 2), 58% (*n* = 29) were from being an occupant in a car crash, 26% (*n* = 13) were pedestrian injuries, and 14% (*n* = 7) and 2% (*n* = 1) were from motorcycle and bicycle injuries, respectively. After road traffic injuries, the next most common injury-related complaints were due to falls. Table 3 depicts the mechanism of injury broken down by age group. The majority of injuries (*n* = 87, 75%) were in children under 5 years of age, and the most common cause of injury in this age group was road traffic injury (*n* = 48, 55%) followed by falls (*n* = 25, 29%). In patients 5 years of age and older, falls (*n* = 12, 41%) and road traffic injuries (*n* = 11, 38%) were approximately evenly represented.

**Table 3:** Types of injury by age

**Types of injury**	**Total*n*= 116,*n*(%)**	**Under 5 years old*n*= 87 (75.0),*n*(%)**	**Five years and older*n*= 29 (25.0),*n*(%)**
Fall	37 (32.0)	25 (28.7)	12 (41.4)
Road traffic injury	59 (50.9)	48 (55.2)	11 (37.9)
Car crash	29 (25.0)	26 (29.9)	3 (10.3)
Pedestrian injury	13 (11.2)	7 (8.0)	6 (20.7)
Motorcycle	7 (6.0)	6 (6.9)	1 (3.4)
Pedal bike/bicycle	1 (0.9)	0 (0.0)	1 (3.4)
Assault/Fight	5 (4.3)	4 (4.6)	1 (3.4)
Burn	5 (4.3)	5 (5.7)	0 (0.0)
Poisoning/overdose	1 (0.9)	1 (1.1)	0 (0.0)
Cut laceration not related to fighting	7 (6.0)	5 (5.7)	2 (6.9)
Other	11 (9.5)	8 (9.2)	3 (10.3)

*Road traffic injury includes car crash, motorcycle crash, pedestrian injuries, and bicycle injury.

Despite the availability of national health insurance [14], 17% (*n* = 30) did not have any kind of health insurance. Of those that were insured (*n* = 137), a large majority (96%) were subscribers to the National Health Insurance Scheme, which is offered by the Ghanaian government.

## Discussion

Among pediatric patients presenting for acute care at the A&E at KATH during the study period, the majority of children (*n* = 116, 66%) presented for injuries, and 43% (*n* = 50) of these injuries were road traffic injuries. In comparison, nearly one-third (32%) of pediatric Emergency Department visits in the US in 2003 were for injuries, of which 28% of patients presented for a fall and 7% presented for a motor vehicle-related injury [15].

Children involved in motor vehicle crashes represented the largest proportion of road traffic injuries presenting to this facility, which is consistent with global trends noted by the World Health Organization (WHO) [3,5]. Worldwide, the majority of road traffic injuries involve children, and this is highest in African and Asian countries [1]. The number of pediatric road traffic injuries is likely to increase with increasing economic growth, improved roads, and increased volume of cars [16] unless a tow-pronged approach that includes improved prevention (i.e., improved seatbelt use, effective speed limits, regulations on drinking and driving) and improved acute care for victims of road traffic injuries is instituted. While data on alcohol consumption were beyond the scope of this analysis, future research should consider the role of alcohol in the severity and timing of motor vehicle crashes. Ghana has a legal blood alcohol concentration limit of 0.8 g/dl for drivers, but it is not effectively enforced [5].

Falls also constituted a large number of injuries among this sample, with one third of injured patients in this analysis presenting for care after a fall. While falls do not account for a large percentage of mortality, according to the WHO they lead to significant morbidity and are the 13th leading cause of disability-adjusted life years (DALY’s) lost worldwide [1]. In Ghana, a previous community-based survey revealed falls to be the leading mechanism of injury in people less than 15 years of age leading to disability [17]. Future studies of acute care facilities that focus on the severity of the fall and subsequent outcomes are needed to inform fall prevention interventions as well as improved understanding of ways to improve outcomes within the resource constraints of the system.

Assessing child maltreatment and non-accidental trauma was out of the scope of this analysis, but there were four subjects under the age of 5 that presented for injury due to an assault or from a fight. The circumstances regarding these assaults are unknown. Future research is needed using a culturally sensitive approach [18] to further investigate the burden of injury due to non-accidental trauma or abuse.

A multidisciplinary approach to the severely injured pediatric patient that includes surgeons (trauma and orthopedics), pediatricians, and trained emergency providers is important to improve the care of these patients. In Kenya and Zambia, practitioners who took a trauma and critical care course (Acute Trauma Care) felt significantly more confident in their ability to take care of critically ill trauma patients after completion of the course compared to prior to the course [19]. Additionally, systems changes such as a triage system implemented by nurses that takes into account injury severity have been shown to decrease wait times for critically ill patients in South Africa [20]. In summary, the high number of patients seeking care for injury within this sample suggests the need for increased resources and training in pediatric acute trauma care.

This study is the first to characterize the pediatric population seeking acute care at an A&E in Ghana and highlights the importance of a curriculum for medical providers that includes trauma and care of the acutely injured child. Historically, much attention has been paid to infectious diseases as a cause of pediatric morbidity and mortality. In 2010, malaria accounted for 26 % of mortality for children under 5 years of age in Ghana [6]. However, malaria morbidity and mortality have decreased substantially because of a multidisciplinary approach as well as training and programs aimed at prevention [21,22]. A similar approach is needed to affect morbidity and mortality due to injuries. Access to increased training and adherence to standard trauma protocols may reduce disability and mortality even without increased resources for the site. For instance, an integral part of reducing all causes of morbidity and mortality in this age group is systematic triage as well as a team approach to major trauma [23]. In addition to the analysis presented here, an ongoing effort by this team of authors has resulted in the institution of a formal triage system at KATH.

In this sample, only half of the injured pediatric patients who could ambulate developmentally (i.e., past infancy) were ambulatory on presentation. This measure is a crude estimate of the severity of illness and injury. Patients presenting for injury were less likely to be ambulatory compared to those presenting for non-injury-related complaints, suggesting that the injuries were not minor. Ambulatory status for excluded patients was not ascertained, but assuming that all the sedated patients were non-ambulatory, inclusion of this cohort might change the odds ratio and this association. If the majority of sedated patients presented for injury-related complaints, this would be an underestimate, and if the majority of sedated patients presented for medical complaints (i.e., fever), this would be an overestimation. Overall, this finding warrants further examination and replication.

Emergency medical transport and trauma systems are underdeveloped in many LMICs such as Ghana. The Ghana Ambulance Service was initiated with ten donated ambulances in 2003 when the Accra soccer stadium collapsed, killing hundreds of people. In this analysis, approximately 20% of patients were transported by ambulance. Additionally, over 50% had traveled more than 1 h for care. Given that the road network in Ghana is underdeveloped and inefficient, time spent travelling may not be reflective of the distance covered. Improved infrastructure and pre-hospital care of these injured patients may provide an opportunity to improve outcomes and decrease secondary disability.

While this study provides novel information regarding pediatric patients seeking acute care, several limitations are worth noting. First, 158 patients did not meet the inclusion criteria and thus were excluded from the study. While being sedated was the number one cause of exclusion, data on the chief complaint were not obtained for excluded patients, and thus it is not known if these sedated patients were presenting for injury or medical reasons. Historically, infectious disease has been a major reason for acute care visits, and future studies should consider adding data from a medical chart review to further understand the characteristics of all pediatric patients presenting for acute care, including those too sedated to speak or participate in survey research. The results of this study need to be interpreted in the context of this large number of excluded participants. Further research to replicate these results is warranted. Additionally, patients were excluded from the study if they were not able to speak Twi, Fante, or English or if translation was not available. However, most of the survey administration was performed by staff able to speak the language of the area, and thus this exclusion was kept to a minimum, preventing bias secondary to inability to speak English. These data were collected within a relatively short time frame at one urban center in Kumasi, Ghana, and could be subject to seasonal or environmental variations. Specifically, results of this study may not be applicable to rural areas in Ghana.

While data were collected systematically over different time frames and days of the week, trends of injury according to time of day were not available. Future studies should note whether patients present with injuries during certain times of the day, which may help with trauma system development and staffing. Due to limitations in the study protocol, there was no information available to the study team regarding the final diagnosis, medical care provided, or final disposition. Similarly, at the time of this survey there was no triage class system, and thus triage categories were not available. There was no availability of initial vital signs taken by the treating physician for use by research staff. Future studies that include detailed data on triage class, discharge diagnosis, and more formal assessment of acuity and patient outcomes are needed.

## Conclusions

Two out of three pediatric patients presenting to the A&E at KATH during the study time period had injury-related complaints. Given the degree of mortality that can result from injury, attention should be placed on the prevention and treatment of childhood injury. This study supports the notion that injury is a common problem and warrants further investigation. Future studies should be aimed at further injury characterization and implementation of sustainable, effective training programs for medical providers, and there should be improved allocation of available resources to the care of the acutely injured pediatric patient. Given the number of non-fatal visits resulting from injury-related complaints, there is a significant need for more injury research and data to help physicians provide care to these patients.

## Competing interests

The author(s) declare that they have no competing interests.

## Authors’ contributions

Drs. Whiteside, Oteng, Carter, Amuasi, Nypaver, and Cunningham contributed to the conception and design of the study. Drs. Whiteside and Oteng and Ms Abban contributed to acquiring data. All authors were involved with interpretation of the data. Drs. Whiteside and Oteng, Ms Abban and Ms Rominski contributed to initially preparing the manuscript. Drs. Carter, Aumuasi, Nypaer, and Cunningham provided critical feedback and editing and contributed to the final manuscript. All authors contributed to the intellectual content and have had a chance to approve the final manuscript to be published.

## References

[B1] Peden MM UNICEF, World Health Organization World report on child injury prevention 2008 Dallas: World Health Organization; UNICEF 26269872

[B2] Injuries and Violence The Facts Geneva, Switzerland: World Health Organization. Bulletin/2010/Bulletin of the World Health Organization. World Health Organization http://whqlibdoc.who.int/publications/2010/9789241599375_eng.pdf. Retrieved 07/02/2011

[B3] World Health Organization Global status report on road safety: time for action 2009 Dallas: World Health Organization

[B4] Patton GC Coffey C Sawyer SM Global patterns of mortality in young people: a systematic analysis of population health data Lancet 2009 374 881 892 10.1016/S0140-6736(09)60741-8 19748397

[B5] Peden M Scurfield R Sleet D Peden M World Report on Road Traffic Injury Prevention 2004 Geneva, Switzerland: World Health Organization 244

[B6] Country Health System Fact Sheet Ghana World Health Organization web site 2010 Available at: http://www.afro.who.int/index.php?Itemid=1840. Accessed November 1

[B7] Ohene SA Tettey Y Kumoji R Injury-related mortality among adolescents: findings from a teaching hospital's post mortem data BMC Res Notes 2010 3 124 10.1186/1756-0500-3-124 20444252 PMC2874566

[B8] http://kma.ghanadistricts.gov.gh/. Accessed January 24, 2011

[B9] London JA Mock CN Quansah RE Priorities for improving hospital-based trauma care in an African city J Trauma 2001 51 747 753 10.1097/00005373-200110000-00021 11586170

[B10] Ghana Demographic and Health Survey 1998, Round 3 2010 Available at: http://www.statsghana.gov.gh/nada/index.php?page=catalog. Accessed September 9, 2010

[B11] Ghana Core Welfare Indicators Questionnaire Survey 2010 Available at: http://www.statsghana.gov.gh/nada/index.php?page=catalog. Accessed Sept 10, 2010

[B12] National Center for Health Statistics & U.S. Health Care Financing Administration The International Classification of Diseases, 9th Revision, Clinical Modification: ICD-9-CM 2000 Washington, D C: US Department of Health and Human Services, Public Health Service

[B13] Vyas S Kumaranayake L Constructing socio-economic status indices: how to use principal components analysis Health Pol Plan 2006 21 459 468 10.1093/heapol/czl029 17030551

[B14] Agyepong IA Adjei S Public social policy development and implementation: a case study of the Ghana National Health Insurance scheme Health Pol Plan 2008 23 150 160 10.1093/heapol/czn002 18245803

[B15] Owens P Zodet M Berdahl T Annual report on health care for children and youth in the United States: focus on injury-related emergency department utilization and expenditures Ambul Pediatr 2008 8 219 240 e17 10.1016/j.ambp.2008.03.032 18644545

[B16] Kopits E Cropper M Traffic fatalities and economic growth Accid Anal Prev 2005 37 169 178 10.1016/j.aap.2004.04.006 15607288

[B17] Mock CN Abantanga F Cummings P Incidence and outcome of injury in Ghana: a community-based survey Bull World Health Organ 1999 77 955 964 10680242 PMC2557773

[B18] Krug EG Dahlberg LL Mercy JA World Report on Violence and Health 2002 Geneva: World Health Organization

[B19] Macleod JB Okech M Labib M Evaluation of trauma and critical care training courses on the knowledge and confidence of participants in Kenya and Zambia World J Surg 2010 35 9 16 21046383 10.1007/s00268-010-0810-z

[B20] Bruijns SR Wallis LA Burch VC A prospective evaluation of the Cape triage score in the emergency department of an urban public hospital in South Africa Emerg Med J 2008 25 398 402 10.1136/emj.2007.051177 18573947

[B21] Kweku M Webster J Adjuik M Options for the delivery of intermittent preventive treatment for malaria to children: a community randomised trial PLoS One 2009 4 e7256 10.1371/journal.pone.0007256 19789648 PMC2748713

[B22] Aponte JJ Schellenberg D Egan A Efficacy and safety of intermittent preventive treatment with sulfadoxine-pyrimethamine for malaria in African infants: a pooled analysis of six randomised, placebo-controlled trials Lancet 2009 374 1533 1542 10.1016/S0140-6736(09)61258-7 19765816

[B23] Molyneux EAS Robertson A Improved triage and emergency care for children reduces inpatient mortality in a resource-constrained setting Bull World Health Organ 2006 84 314319 10.2471/blt.04.019505 PMC2627321 16628305

